# VHL and Hypoxia Signaling: Beyond HIF in Cancer

**DOI:** 10.3390/biomedicines6010035

**Published:** 2018-03-19

**Authors:** Jing Zhang, Qing Zhang

**Affiliations:** 1 Department of Pathology and Laboratory Medicine, Lineberger Comprehensive Cancer Center, UNC-Chapel Hill, Chapel Hill, NC 27599, USA; 2 Department of Pharmacology, UNC-Chapel Hill, Chapel Hill, NC 27599, USA

**Keywords:** VHL, Hypoxia, ccRCC

## Abstract

Von Hippel-Lindau (VHL) is an important tumor suppressor that is lost in the majority of clear cell carcinoma of renal cancer (ccRCC). Its regulatory pathway involves the activity of E3 ligase, which targets hypoxia inducible factor α (including HIF1α and HIF2α) for proteasome degradation. In recent years, emerging literature suggests that VHL also possesses other HIF-independent functions. This review will focus on VHL-mediated signaling pathways involving the latest identified substrates/binding partners, including N-Myc downstream-regulated gene 3 (NDRG3), AKT, and G9a, etc., and their physiological roles in hypoxia signaling and cancer. We will also discuss the crosstalk between VHL and NF-κB signaling. Lastly, we will review the latest findings on targeting VHL signaling in cancer.

## 1. Introduction

The incidence of kidney cancer has been increasing for decades, causing 134,000 deaths annually worldwide and accounting for an estimated 62,700 new cases as well as 14,240 deaths in the United States of America (USA) in 2016 [1,2,3]. Clear cell carcinoma of renal cancer (ccRCC) makes up approximately 70% of all renal malignancies and harbors up to 92% of Von Hippel-Lindau (*VHL*) inactivation [2,4,5,6,7]. Despite a significant mutation heterogeneity within ccRCC, *VHL* inactivation occurs ubiquitously across all tumor regions [2,8]. VHL is a tumor suppressor in ccRCC that regulates its substrates by forming a complex with elongins B and C (termed “VBC”), which are key components of an E3 ubiquitin ligase complex that is crucial for VHL function [9,10,11,12]. It is well known that this E3 ligase complex targets hypoxia inducible factor α (HIFα, including HIF1α and HIF2α) for ubiquitination and degradation, which contributes substantially to the transforming phenotype of renal cancer [13,14]. Further research has shown that VHL interacts with HIF1α through the hydroxylation of defined HIF1α proline residues (prolines 402 and 564) by members of the EglN family of iron- and 2-oxoglutarate-depedent dioxygenases (EglN1, EglN2, and EglN3) [15]. Therefore, the loss of VHL leads to HIFα accumulation and translocation into the nucleus, which subsequently activates the transcription of HIF target genes that are involved in critical oncogenic pathways, including angiogenesis (e.g.,Vascular endothelial growth factor ((*VEGF*)), glycolysis and glucose transport (e.g., Glucose transporter type I (*GLUT1*)), and erythropoiesis (e.g., Erythropoietin (*EPO*)) [16,17,18,19,20]. Advances in HIF regulation have been recently reviewed extensively by Choudhry et al. [21].

Although the degradation of HIFα is important for the tumor suppressor function of VHL, growing evidence suggests that additional VHL substrates exist. For example, the existence of uncharacterized VHL substrates other than HIF may account for different subtypes of VHL disease (Type 2A, 2B versus 2C) [22,23,24]. The VHL-mediated assembly of intercellular junctions is through HIF-independent mechanisms in VHL-deficient ccRCC [25]. Also, the AKT, the Notch signaling cascade, and the NF-κB signaling pathway are constitutively active upon *VHL* loss in ccRCC [26,27,28]. Our colleagues have reviewed these recently identified VHL substrates/binding partners extensively elsewhere [29]. In this review, we focus on VHL-regulated signaling pathways involving newly identified substrates/binding partners that may play important roles in hypoxia signaling beyond HIF.

## 2. Hypoxia/VHL and Lactate–NDRG3–Raf–ERK Axis

Lee et al. identified NDRG3 as a substrate of EglN1/VHL through Flag-mediated immunoprecipitation of EglN1, followed by mass spectrometry. Under normoxia, NDRG3 is ubiquitinated and degraded via the EglN1-VHL signaling pathway. NDRG3 accumulates under hypoxia by binding to lactate, and further interacts with c-Raf for the activation of the Raf-ERK pathway, thereby contributing to hypoxic cell growth and angiogenesis. NDRG3 remains very stable once it is bound to lactate, even when cells are reoxygenated, which suggests that lactate–NDRG3–Raf–ERK axis-induced responses contribute to the maintenance of tumor progression under prolonged hypoxic conditions. This study identified NDRG3 as the hypoxia-inducible lactate sensor that provides crucial hypoxia signaling of oxygen-dependent regulation in an HIF-independent manner; therefore, a combinatorial targeting of both HIF and NDRG3 might be a highly effective therapy in cancer [30,31].

It is important to point out that this study provides an additional model (besides the ROS–EglN–HIF axis) of wiring metabolism to signal the transduction cascade for downstream gene regulation and corresponding cell responses. Altered metabolism is one of the hallmarks of cancer, with the underlying mechanism potentially revealing the vulnerability of the cancer [32,33]. The abundance of nutrients (e.g., glucose, glutamine, and lipid) fluctuates significantly along with oxygen availability or VHL status [34]. For example, Hakimi et al. deciphered the metabolic atlas of ccRCC by using metabolomics profiling, and characterized broad shifts in the central carbon metabolism, the one-carbon metabolism, and the antioxidant response in ccRCC patients [35]. Therefore, how these altered metabolic pathways are wired to regulate cellular adaptation to the harsh tumor growth environment needs to be further investigated in the future. 

## 3. Hypoxia/VHL–AKT–mTOR Axis

PI3K/AKT/mTOR signaling is a well-established cell growth regulatory pathway. AKT phosphorylation at Thr308 and Ser473 increases its kinase activity to stimulate its downstream mTORC1 activation, leading to an increase in the activity of various anabolic, biosynthetic pathways for cell growth and proliferation [36]. The PI3K/AKT/mTOR pathway is altered with hypoxia/VHL. For example, Polytarchou et al. reported that the AKT pathway is activated in hypoxic conditions, which promotes tumor resistance to hypoxia through the induction of miR-21 [37]. Phosphorylated, active AKT is recruited to mitochondria under hypoxic conditions, whereby PDK1 is phosphorylated at T346, which switches tumor metabolism to glycolysis and maintains the redox balance needed for tumor cell survival and proliferation [38]. Guo et al. showed that AKT is activated to promote tumorigenesis in cells that lack oxygen or functional VHL. This was a mechanistic study that demonstrated that VHL binds to hydroxylated AKT induced by EglN1 and inhibits its phosphorylation and kinase activity in an HIF-independent and prolyl-hydroxylation-dependent fashion. Collectively, the intersection between hypoxia/VHL and AKT signaling involves multiple steps, including microRNA regulation, subcellular compartment change, and post-translational regulation (hydroxylation). Recently, Zhang et al. revealed a pan-cancer proteogenomic atlas for PI3K/AKT/mTOR changes across over 10,000 human cancers, supporting the notion that VHL mutations are associated with highly active AKT/mTOR signaling [39]. These studies provide the therapeutic strategy of targeting PI3K/AKT/mTOR in VHL-deficient ccRCC and in hypoxic tumors to overcome drug resistance.

Indeed, two Food and Drug Adminstration (FDA)-approved mTOR inhibitors, temsirolimus and everolimus, showed improved clinical outcomes and provided additional options for difficult-to-treat cancers. As the targeted therapies are growing so rapidly, an urgent challenge is how to design the most effective combinatory treatments to prevent the emergence of escape mechanisms [2,3].

## 4. VHL–NF-κB Axis

NF-κB is one of the key regulators of inflammation. The NF-κB family, as a transcription factor, consists of p65 (RelA), c-Rel, RelB, p50/p105 (NF-κB1), and p52/p100 (NF-κB2). In a resting cell, they exist as homodimers or heterodimers preferentially bound to the IkB family, which prevents DNA-binding by NF-κB activation. Extracellular or intracellular signals initiate NF-κB activation, such as pro-inflammatory cytokines (e.g., tumor necrosis factor (TNF)), the recognition of extracellular or intracellular pathogens (e.g., toll like receptors (TLRs)), and cell stressors (e.g., reactive oxygen species). More direct, aberrant protein expression also activates the NF-κB pathway (e.g., VHL loss) [26,40]. Upon stimulation, IκB is degraded or processed by proteasomes, which release NF-κB and subsequently cause NF-κB activation and its downstream gene transcription [41,42,43].

NF-κB hyper-activation can promote resistance to chemotherapy or cytokine treatments and correlates with a worse outcome in ccRCC [44,45,46]. Previous studies by others showed that hypoxia can induce NF-κB activation through the EglN2 negative regulation of IKKβ. NF-κB activity is also increased in VHL-deficient ccRCC [26,40,47]. ccRCC cells with VHL inactivation are resistant to TNF cytotoxicity, and their sensitivity to TNF can be restored by reconstituted wild-type VHL in these cells, at least partially through suppressing the NF-κB-dependent anti-apoptotic pathway [40]. Nevertheless, the way in which VHL regulates NF-κB activity remains unclear. One underlying mechanism is that VHL functions as an adaptor that promotes the inhibitory phosphorylation of the NF-κB agonist, Card9, by enhancing the interaction between Card 9 and CK2 in an HIF-independent fashion [26,40]. As a substrate recognition unit of an E3 ubiquitin ligase complex, it remains to be determined as to whether there are VHL substrates regulating NF-κB pathway activity in cancer.

The intersection between inflammation and cancer has recently blossomed. It has been demonstrated that tumor-promoting inflammation contributes to the acquisition of multiple hallmark chemicals that are needed for cancer initiation and progression, including growth factors that maintain cell proliferation (e.g., VEGF); survival factors that inhibit cell apoptosis (e.g., BCL2); extracellular matrix-modifying proteins that promotes angiogenesis, invasion, and metastasis (e.g., MMP); and other hallmark-promoting chemicals [32,48]. Cancer-promoting inflammation has been well established as a promising target [49,50,51]. Besides NF-κB, there are AP-1, CEBP, and JunB, etc., which are involved in both inflammation and hypoxia/VHL-related cancer progression [52,53]. The underlying molecular mechanisms of how hypoxia or VHL is linked to inflammation need to be clarified.

## 5. EglN3–VHL–EPOR Axis

Red blood cell production increases oxygen-carrying capacity and enables the body to survive harsh hypoxic conditions. For example, erythropoietin (EPO) stimulates erythropoietin receptor (EPOR)-JAK2-STAT5 signaling, which is responsible for cell proliferation and terminal differentiation into mature, oxygen-carrying red blood cells [54,55]. EPO is regulated by extrinsic EglN1-VHL-HIF signaling [56,57,58]. Heir et al. found that EPOR turnover is regulated by oxygen availability through intrinsic EglN3-VHL-EPOR signaling that is independent of HIF. Specifically, the proline 419 and 426 residues within the cytoplasmic region of EPOR are hydroxylated by EglN3, followed by VHL binding for polyubiquitination and proteasome degradation [59]. However, the functional roles of the EglN3–VHL–EPOR axis in hypoxic or VHL loss-of-function diseases remain to be determined in the future.

## 6. VHL–B-Myb Axis

B-Myb is a transcription factor that regulates the cell cycle and chromosomal condensation and stability [60,61]. Okumura et al. showed that B-Myb is targeted by VHL for ubiquitination and proteasome degradation, which can be prevented by the VEGF/platelet derived growth factor (PDGF)-induced T15 phosphorylation of B-Myb [62]. The authors showed that B-Myb depletion increases tumor growth by regulating different downstream genes from HIF and can antagonize HIF-dependent tumorigenesis in an HIF-independent manner. The detailed molecular mechanism, however, needs to be clarified [62].

## 7. Other VHL Downstream Regulators

VHL may also play multiple roles in other various cell responses/diseases, including synaptogenesis (by targeting Filamin A (FLNA)), centrosome function (by targeting Cep68), and antiviral immune defense (by targeting mitochondrial antiviral signaling protein (MAVS)) through its E3 ubiquitin ligase protein activity in prolyl hydroxylation that can occur in dependent or independent pathways [63,64,65]. Specifically, PP5 and AURKA are targeted by VHL through oxygen-independent regulation [66,67]. MAVS and KLF4 are VHL substrates, but whether these regulations are dependent on oxygen availability is unknown [63,68]. The oxygen/VHL-mediated ubiquitination and proteasome degradation of proteins involve EGFR, atypical protein kinase C, Sprouty 2, β-adrenergic receptor II, Myb-binding protein 160, RPB1, RPB7, Cep68, Interleulin-32β, CERKL, FLNA, and ERK5/BMK1 [64,65,69,70,71,72,73,74,75,76,77,78,79]. In addition, VHL transcriptionally regulates aldehyde dehydrogenase 2 (*ALDH2*) through the direct activation of transcription factor HNF-4α in an HIF- and VHL E3 ligase-independent fashion, which contributes to the sensitivity of ccRCC cells to anthracycline treatment [80]. Although previous research suggested that ccRCC cell lines display constitutive active Notch signaling that is independent of VHL/HIF signaling [81], a very recent study demonstrated that VHL mutations affect vessel branching and maturation via Notch [28], though the underlying mechanism remains unknown. These substrates/regulators may provide new clues for a deeper understanding of the physiological and pathological functions of VHL.

## 8. VHL and Epigenetic Regulation

Most of these studies focus on the role of VHL/HIF activation on the proximal promoter of target genes. In recent years, the role of HIF on distant promoters and enhancers has gained more attention. For example, by using a chromosomal conformation assay with high-resolution analyses, Platt et al. recently found the long-range interactions between intergenic HIF binding regions and promoters of canonical *HIF* target genes [82]. However, these interactions did not seem to be affected by HIF activation through either hypoxia or VHL loss. HIF2α was also reported to bind distant enhancers for Myc and Cyclin D1 to promote their transcription in ccRCC [83,84]. Yao et al. used paired primary patient tumor and normal samples, ccRCC cell lines, and normal kidney cell lines, followed by an extensive profiling of histone markers including H3K27Ac (marks active enhancer), H3K4me3 (marks active transcription in proximity to promoters), and H3K4me (marks enhancers) in ccRCC [85]. It is worth mentioning that they confirmed that 90% of tumors carried VHL loss-of-function mutations. First, they found that tumor-specific gained enhancers were associated with important oncogenic signaling and the hallmarks of ccRCC, including HIF1α signaling and pro-angiogenesis pathways. Specifically, VEGFA, CXCR4, GLUT1, HK2, and PFKFB3 were all associated with gained enhancers. High resolution Capture-C also confirmed the distant interaction (100 kb) between VEGFA enhancer and its transcription starting site (TSS). In addition, they also identified the addition of super-enhancers in several potential novel oncogenes with VHL loss, including ZNF395. More importantly, their research demonstrated that VHL restoration largely leads to a diminished intensity in gained enhanced H3K27ac, indicating that these enhancers represent VHL-dependent epigenetic regulation in ccRCC. Furthermore, VHL-loss-mediated enhancer remodeling is mainly mediated by HIF2α-HIF1β-p300 signaling complexes. Accumulatively, this important research showed that the HIF2α signaling complex may play an important role in VHL-mediated effects on enhancers that are important for ccRCC carcinogenesis. 

VHL-loss-induced HIF regulates the expression of many histone lysine demethylases (KDMs) [86,87,88,89]. Therefore, it is likely that ccRCC, mostly with VHL loss, would demonstrate a distinctive histone modification. By using a multiplexed and high-resolution quantitative mass spectrometry approach, Chakraborty et al. found that ccRCC tumors with VHL loss preferentially upregulate H3K27ac and H3K27me0/me1 levels [90]. More strikingly, VHL loss leads to the depletion of H3K27me3 due to increased H3K27 demethylase KDM6B activity in ccRCC cells. Since KDM6B is an *HIF* target gene [91], this study suggests that HIF-induced histone demethylase activity renders VHL-null ccRCC sensitive to H3K27 methyltransferase EZH1 inhibition. This important study will likely stimulate the development of EZH1-specific inhibitors for the treatment of ccRCC.

Hypoxia has been reported to upregulate G9a protein levels while not affecting their mRNA, indicating a potential post-transcriptional regulation of G9a by hypoxia [92]. However, the underlying mechanism remains unclear. In this latest study, Casciello et al. reported that G9a undergoes PHD1/EglN2-mediated prolyl hydroxylation, followed by its degradation via VHL [93]. Upon the treatment of hypoxia, G9a hydroxylation is inhibited and VHL cannot bind with G9a, which leads to its upregulation of protein levels. G9a is a major enzyme that converts unmodified H3 lysine 9 (H3K9) such that it becomes dimethylated. As a result, G9a accumulation leads to increased H3K9 dimethylation on a specific set of genes (including ARNTL and HHEX), which are essential for the function of tumor suppression under hypoxia. A G9a methyltransferase inhibitor, UNC0642, was used to test its efficacy in a syngeneic luminal breast cancer model, and it significantly reduced the tumor growth in vivo [94]. In summary, this paper showed that hypoxia, by regulating G9a protein levels and through the H3K9 dimethylation of key tumor suppressor promoters, contributes to an increased breast tumorigenesis. In theory, VHL loss is expected to increase G9a levels in ccRCC. It remains to be determined as to whether G9a can be used as a potential therapeutic target in ccRCC.

It is worth noting that the loss of VHL was reported to induce DNA replication stress and cause the accumulation of DNA damage that constrains cell proliferation and oncogeneic transformation. However, the additional loss of the chromatin remodeling factor, PBRM1, in ccRCC, rescues this replication stress through the modulation of H3K9me3, therefore contributing to the oncogenic transformation in ccRCC [95]. It remains to be determined as to how VHL loss affects DNA replication stress.

## 9. Targeting VHL Signaling in ccRCC

While HIF1α serves mainly as a tumor suppressor in kidney cancer [96,97], HIF2α stabilization, as a result of VHL loss, is sufficient and necessary in the promotion of kidney tumor growth [98]. Therefore, HIF2α serves as an important therapeutic target in kidney cancers that are related to *VHL* loss. However, HIF2α has been called “undruggable” [99]. Previously, most of the therapeutic targeting strategies in VHL-null ccRCC have focused on using mTOR inhibitors (such as everolimus or temsirolimus) to inhibit HIF translation. Many therapeutic targeting strategies have also focused on the use of kinase inhibitors (including bevacizumab, sunitinib and sorafenib) to inhibit HIF downstream targets, including VEGF and PDGF, which has been reviewed extensively in previous publications [3]. Based on a high-throughput screening (HTS) and efforts in medicinal chemistry, recent studies have discovered a highly selective HIF2α antagonist that does not affect HIF1α signaling [100]. Follow-up studies have been carried out to test the drug-like HIF2α chemical, PT2399, in ccRCC cell lines, cell-line-based orthotopic xenograft mouse models, a VHL-deficient metastatic setting, as well as RCC tumorgraft-bearing mice [101,102]. PT2399 selectively inhibits HIF2α (but not HIF1α) binding with ARNT, an essential co-factor for the transactivation of downstream target genes by HIF2α for oncogenesis. As a result, PT2399 efficiently blocked ccRCC cell proliferation in a 3-D culture, tumor growth, and metastatic potential in vivo. More importantly, PT2399 displayed superior efficacy and better tolerability compared to current standard ccRCC therapy, such as sunitinib [102]. Therefore, the findings from these papers reveal the first potent HIF2α inhibitor that can be optimized and potentially used in the treatment of ccRCC. PT2385, an analogue of PT2399, is now being tested in the Phase I clinical trial [103]. It is important to point out that not all VHL-deficient ccRCC cell lines demonstrate sensitivity to treatment with PT2399. It has also been shown that p53 mutations may be associated with an acquired resistance to PT2399 treatment [101]. Further investigations should be carried out to identify predictive biomarkers for HIF2α inhibitors in ccRCC, which may help validate these inhibitors in the clinical setting.

Although targeted therapies have been proven to be effective for certain tumors which express gain-of-function oncoproteins, therapeutic resistance eventually develops. It is possible to identify second-site targets that, when combined with a tumor specific mutation, cause a specific cancer cell death without affecting normal cells. This is called “synthetic lethality”, which has emerged as an important concept in cancer therapies [104,105]. Tumor-specific genetic alterations (such as *VHL* loss) reveal not only the biological changes that drive tumor progression but also vulnerabilities that can be exploited therapeutically. Since up to 90% of kidney tumors harbor VHL functional loss, it remains very attractive to identify synthetic lethality partners in VHL-loss kidney cancer while sparing normal cells.

By screening a small molecular library, Turcotte et al. identified STF-62247 as a promising compound that is selectively toxic toward VHL-deficient ccRCC cells [106]. Mechanistically, they identified that STF-62247 induces cell toxicity through autophagy and HIF-independent signaling. This discovery has stimulated a series of follow-up studies which focus on VHL deficiency as a target in ccRCC. In addition, there has been continuous interest in targeting VHL and HIF-dependent signaling in ccRCC. For example, STF-31 was found to specifically target VHL-loss ccRCC cells because it inhibits glucose uptake through Glut1, which is a canonical target of HIF signaling [107]. Chromomycin A3 was also identified to be toxic to VHL-deficient ccRCC cells because it acts as an HIF-dependent cytotoxin [108]. In addition, Rho-associated kinase 1 (ROCK1) was identified to be synthetically lethal with a VHL loss in an HIF-dependent manner [109]. The latest research on the selective toxicity of EZH1 inhibitors on VHL-null ccRCC cells also showed their dependency on HIF signaling [90]. On the other hand, there has been accumulating evidence suggesting that some of the synthetic lethality partners in VHL-loss tumors may not depend on HIF signaling. For example, a focused shRNA library screening identified that CDK6, MET, or MEK1 shRNAs may be selectively toxic to VHL-deficient ccRCC cells, but the effect is mediated in an HIF-independent fashion [110]. In addition, a highly sensitive high-throughput imaging-based platform with 12,800 small molecules identified homoharringtonine (HHT), an FDA-approved drug that may be used to treat chronic myeloid leukemia (CML), as a synthetic lethality partner in VHL-loss tumors [111]. However, it remains to be determined as to whether HHT affects HIF signaling in this setting. These new emerging targets/agents await to be further tested in potential future clinical trials.

## 10. Perspectives

Recently, proteolysis targeting chimeras (PROTACs) emerged as the new and powerful technology that can be used to target “undruggable” proteins for degradation [112]. PROTACs contain one moiety that binds an E3 ligase linked to another moiety that binds a desired cellular target protein. This induced proximity results in the ubiquitination of the target, followed by its degradation at the proteasome. VHL has been widely used as the E3 ligase adaptor for PROTAC. For example, there was a recent study that identified a specific TBK1 degrader by using VHL as the E3 ligase adaptor in PROTACs, which achieves the specific degradation of TBK1, but not its close family member IKKε [113]. Therefore, it is promising that VHL can be used as a tool to degrade the “undruggable” targets in cancer that may achieve efficient therapeutic effects in cancer therapy.

## 11. Conclusions

**Figure 1:**
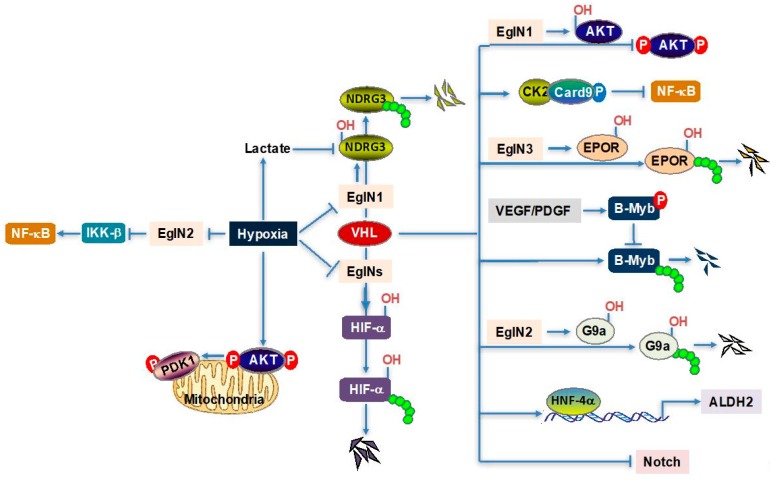
Schematic of multi-functionality of VHL in hypoxia signaling and cancer.

While HIF and HIF-dependent signaling remain to be therapeutically interrogated in cancers with VHL loss, during the past few years, there have been several emerging studies that studied the role of VHL in the regulation of HIF-independent pathways. VHL can not only regulate other substrates (including NDRG3, G9a, and EpoR) through its canonical E3 ligase function, but also can be used as an adaptor protein that recruits the phosphatase pathway to mitigate constitutive AKT phosphorylation (Figure 1). It is an exciting time to study the multi-functionality of VHL in hypoxia signaling and cancer, which may yield additional therapeutic targets in cancer and other diseases.
